# Biomechanics show stem cell necessity for effective treatment of volumetric muscle loss using bioengineered constructs

**DOI:** 10.1038/s41536-018-0057-0

**Published:** 2018-10-10

**Authors:** Marco Quarta, Melinda J. Cromie Lear, Justin Blonigan, Patrick Paine, Robert Chacon, Thomas A. Rando

**Affiliations:** 1 Department of Neurology and Neurological Sciences, Stanford University School of Medicine, Stanford, CA 94305 USA; 2 Paul F. Glenn Laboratories for the Biology of Aging, Stanford University School of Medicine, Stanford, CA 94305 USA; 3 Center for Tissue Regeneration, Restoration and Repair, Veterans Affairs Hospital, Palo Alto, CA 94036 USA; 4 Molecular Medicine Research Institute, Sunnyvale, CA 94085 USA

## Abstract

Despite the regenerative capacity of muscle, tissue volume is not restored after volumetric muscle loss (VML), perhaps due to a loss-of-structural extracellular matrix. We recently demonstrated the structural and functional restoration of muscle tissue in a mouse model of VML using an engineered “bioconstruct,” comprising an extracellular matrix scaffold (decellularized muscle), muscle stem cells (MuSCs), and muscle-resident cells (MRCs). To test the ability of the cell-based bioconstruct to restore whole-muscle biomechanics, we measured biomechanical parameters in uninjured muscles, muscles injured to produce VML lesions, and in muscles that were injured and then treated by implanting either the scaffolds alone or with bioconstructs containing the scaffolds, MuSCs, and MRCs. We measured the active and passive forces over a range of lengths, viscoelastic force relaxation, optimal length, and twitch dynamics. Injured muscles showed a narrowed length-tension curve or lower force over a narrower range of muscle lengths, and increased passive force. When treated with bioconstructs, but not with scaffolds alone, injured muscles showed active and passive length-tension relationships that were not different from uninjured muscles. Moreover, injured muscles treated with bioconstructs exhibited reduced fibrosis compared to injured muscles either untreated or treated with scaffolds alone. The cell-based bioconstruct is a promising treatment approach for future translational efforts to restore whole-muscle biomechanics in muscles with VML lesions.

## Introduction

Volumetric muscle loss (VML) is a severe traumatic injury or surgery excision that limits the ability of the patient to perform activities of daily living, resulting in significant functional loss.^1^ Despite the regenerative capacity of muscle after other types of injury, tissue volume is not restored after VML, perhaps due to a loss-of-structural extracellular matrix.^2^ In an attempt to restore the lost muscle tissue, some tissue-engineered therapeutics have been tested, with some improvement in active force.^3,4^

To restore functional motion, a treated muscle must recreate the native whole-muscle biomechanics, which arise from muscle architecture. In a recent report, we achieved force recovery to near uninjured levels in an animal model of VML by implanting tissue-engineered “bioconstructs” comprised of extracellular matrix scaffolds injected with satellite cells (or muscle stem cells (MuSCs) and other muscle-resident cells (MRCs).^5^ To test the ability of the cell-based bioconstruct treatment to restore whole-muscle biomechanics, we measured in the current studies the active and passive forces over a range of lengths, viscoelastic force relaxation, optimal length, and twitch dynamics, as well as fibrosis of the injured muscles, whether treated or untreated.

## Results

Biophysical measurements, such as active and passive force analysis, are the gold standard for assessments in skeletal muscle physiology. With this aim in mind, we decided to investigate the biomechanical properties of muscles with VML injuries that received: (1) injury but no-treatment; (2) treatment with extracellular matrix-derived scaffold; and (3) treatment with scaffolds that were seeded with MuSCs and MRCs. In each case, we compared the results to those from healthy, non-injured muscles.

**Fig. 1:**
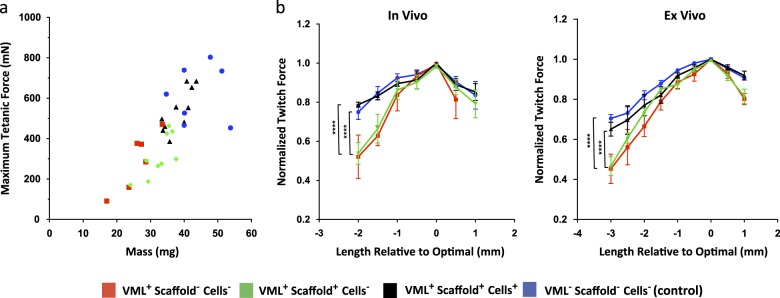
**a** Relationship between maximum isometric tetanic force measured ex vivo and muscle mass in mouse TAs. Control muscles are designated as “VML^−^ Scaffold^−^ Cells^−^”. In the VML injury groups, muscles were partially excised and left untreated (VML^+^ Scaffold^−^ Cells^−^) or implanted with either a tissue-engineered bioconstruct comprising of scaffold alone (VML^+^ Scaffold^+^ Cells^−^) or with scaffold and muscle stem cells, and muscle-resident cells (VML^+^ Scaffold^+^ Cells^+^). “VML^+^ Scaffold^+^ Cells^+^” muscles showed proportionally increased mass and, statistically significant, force (Table 1), consistent with functional active stress generation in the newly formed tissue. **b** Active twitch force across a range of muscle lengths. (Left panel) In vivo measurements. (Right panel) Ex vivo measurements. The “VML^+^ Scaffold^−^ Cells^−^” muscles have narrowed length-tension curves (comparisons between VML^+^ Scaffold^+^ Cells^−^ group and VML^+^ Scaffold^+^ Cells^+^ or VML^−^ Scaffold^−^ Cells^−^ groups; *p* < 0.0001). The length-tension curves of the “VML^+^ Scaffold^+^ Cells^+^” muscles were restored with treatment, meaning that a greater fraction of the maximum force was generated over a broader range of muscle lengths. No improvement was observed with “VML^+^ Scaffold^+^ Cells^−^” treatment. The curve from each muscle was normalized by its own maximum force and centered at optimal length. Symbols are the mean forces and error bars represent SEM (*n* = 6–9 muscles per group)

First, we analyzed active forces to assess the contractile capacity of muscles with VML injuries, with or without treatments. Muscles from all four groups fell along a line of proportionally increasing force and mass, with the VML group treated with scaffolds and cells falling between the untreated VML group and the VML group treated with scaffold only, positioned on the lower end, and the uninjured control group on the upper end (Fig. 1a). Maximum tetanic forces decreased in the untreated VML group and in the VML group treated with scaffold alone compared to uninjured muscles, but did not decrease in the VML group treated with scaffold and cells (Table 1). These results suggest that treatments based on scaffolds and cells, but not treatments based on scaffolds alone, can restore to levels similar to healthy muscles the loss-of-force production that results from VML injuries in our model. Conversely, specific tetanic forces did not show any difference among the four groups, suggesting no dynamic changes such as a shift in myofiber types (Table 1). Next, we analyzed the length-tension curve. We found that it followed the expected shape with a downward curve and a maximum in the midrange (Fig. 1b). The active force-length narrowed with VML injury (in vivo *p* = 0.04; ex vivo *p* = 0.04), and was restored in the VML group treated with scaffolds and cells but not in the VML group treated with scaffold only.

**Table 1:** Muscle biophysics and biomechanics

Condition	Maximum tetanic force (mN)	Specific tetanic forces (N/mm^2^)	Optimal length (mm)	Time to peak force in vivo (ms)	Time to half-relaxation in vivo (ms)
Untreated					
VML^+^ Scaffold^−^ Cells^−^	292.30 ± 143.73	0.0108 ± 0.0012	12.9 ± 0.3	33.4 ± 1.5	26.2 ± 3.2

Treated					
VML^+^ Scaffold^+^ Cells^−^	312.27 ± 106.45	0.0108 ± 0.0029	12.7 ± 0.3	34.3 ± 1.4	27.3 ± 3.3
VML^+^ Scaffold^+^ Cells^+^	539.96 ± 105.45**	0.0111 ± 0.0015	12.5 ± 0.3	36.2 ± 1.3	37.4 ± 3.5*

Uninjured					
VML^−^ Scaffold^−^ Cells^−^	620.21 ± 142.04**	0.0112 ± 0.0003	13.9 ± 0.3*	33.9 ± 0.8	36.5 ± 1.2*

Maximum tetanic force (data from experiments shown in Fig. 1a) was reduced in the untreated VML group (VML^+^ Scaffold^−^ Cells^−^) and in the VML group treated with scaffold only (VML^+^ Scaffold^−^ Cells^−^) compared to the VML group treated with scaffold plus cells (VML^+^ Scaffold^+^ Cells^+^) (or to the uninjured control group). No differences were found between the groups in normalized tetanic force. The optimal muscle length differed between the VML group treated with scaffold plus cells (VML^+^ Scaffold^+^ Cells^−^) and the uninjured control group (VML^−^ Scaffold^−^ Cells^−^), despite the similarity of the length-tension curves for these groups. The untreated VML group (VML^+^ Scaffold^−^ Cells^−^) had a faster recovery as measured by time from peak force to half the peak force. No differences were found between the groups in time to peak force. Values are means ± SEM. Groups were compared using one-way ANOVA followed by post hoc Dunnett’s test. Asterisks indicate significant adjusted *p*-values from comparison to the uninjured group (“VML^−^ Scaffold^−^ Cells^−^)” from the post hoc Dunnett’s test with *α* = 0.05

**Fig. 2:**
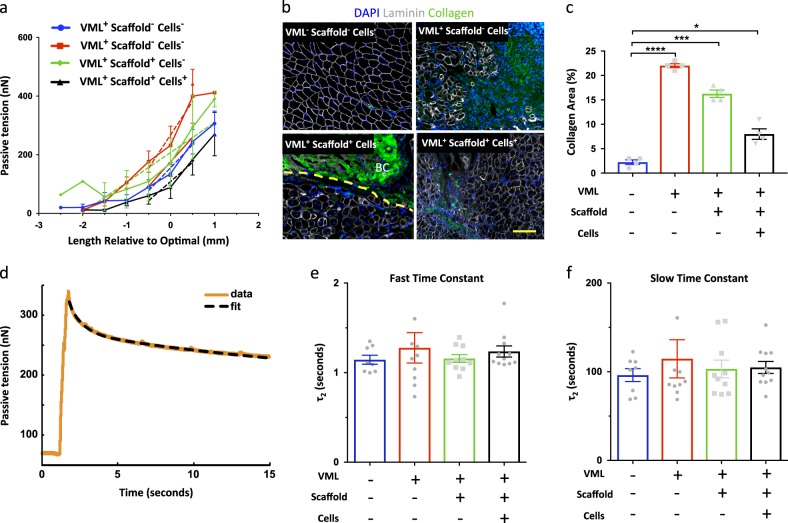
**a** Passive tension across a range of muscle lengths. Passive tension was measured at increasing lengths after 2 min of viscoelastic relaxation at each length. Horizontal axis was offset to the optimal length. Regression lines through each curve in the range L_o_−0.5 to +0.5 were compared. The “VML^+^ Scaffold^+^ Cells^+^” group had lower passive tension than the “VML^+^ Scaffold^−^ Cells^−^” group (*p* < 0.01). The treatment restored the low levels of passive tension observed in the “VML^−^ Scaffold^−^ Cells^−^” uninjured control group. Symbols are the means and error bars represent SEM (*n* = 8–11 muscles per group). **b** Representative immunofluorescence images of cross-sections of VML-injured TA muscles treated with cell-based tissue engineering compared to no-treatment and no-VML. The broken yellow line highlights the border between the regions of regenerative fibrosis below and the dense bioconstruct (BC) above. Collagen I (green); Laminin (white); DAPI (blue) (scale bar = 200 μm). **c** Quantification of immunofluorescence staining against Collagen I protein in regions not occupied by transplanted scaffolds in cross-sections of TA muscles (*n* = 4). **d** Force-relaxation test in response to stepwise increase in length and viscoelastic time constants. A stepwise length increase of 0.5 mm was applied, and the passive force recorded. **e**, **f** A two-exponential function was fit to the curve and the two resulting time constants reported. No difference was observed in viscoelastic relaxation time constants among the groups in either *τ*_1_ (*p* = 0.72) or *τ*_2_ (*p* = 0.68). Bars are the means and error bars represent ± SEM (*n* = 8–11 muscles per group)

VML injuries result in extended fibrosis and increased tissue stiffness.^6,7^ To investigate these aspects, we first analyzed the passive tension curves and we found that they followed the expected shape of a nonlinear spring (Fig. 2a). Substantial passive tension was present at the optimal length in all groups. As expected, passive tension was higher with VML (*p* < 0.01). However, it was restored to uninjured levels by the treatment with both scaffold and cells but not with treatment with scaffold only. These results suggest that treating VML with scaffold plus cells can restore active forces to physiological optimal levels in VML injuries.

To confirm that changes in passive tension correlated with increases fibrosis, we performed immunostaining for Collagen I as a surrogate for the area of fibrosis in the muscle. We found in regions not occupied by transplanted scaffolds that fibrotic tissue was widely distributed in cross-sections of injured muscles of the untreated injured group, with Collagen I deposition mostly located around the VML injury. Conversely, the VML group treated with scaffold plus cells showed reduced fibrosis compared to the untreated VML group and the VML group treated with scaffold only. The uninjured control group showed no evident fibrosis (Fig. 2b, c). Taken together, these results suggest that treatment with scaffold plus cells is more effective compared to treatment with scaffold alone in reducing fibrosis and preventing passive tension in VML injuries.

Next, we decided to look for changes in physiological muscle function, such as contractile kinetics and passive relaxation. First, we analyzed viscoelastic relaxation curves and we found that they were well fit by a two-phase exponential (Fig. 2d). No significant differences among the means were found for *τ*_1_ or *τ*_2_ (Fig. 2e, f). Viscoelastic time constants are similar to those of titin unfolding in myofibrils^6^ and fiber sliding in tendon.^8^

We then measured muscle optimal lengths and we found differences among the means (*p* = 0.05) (Table 1). In post hoc pairwise comparison of all four groups, the lowest *p*-value was between the uninjured control group and the VML group treated with scaffold plus cells (*p* = 0.06). The means of those two groups differed by 10%.

Finally, we analyzed the twitch dynamics. As expected, twitch rise times were consistent with fast twitch muscle^9^ and were not different between conditions (Table 1). The time to half relaxation was not different between the uninjured control group and the VML groups treated with scaffold plus cells or with scaffold only. The untreated VML group had a lower time to half relaxation, possibly due to a greater passive stiffness that facilitates the return of energy during relaxation.

In conclusion, VML injuries result in significant changes of muscle function and structure, compromising active and passive forces and generating extended fibrosis. Cell-based engineered treatments can restore, at least partially, these defects to physiological levels, suggesting promising strategies to design treatments for VML.

## Discussion

The restoration of the active and passive tension properties demonstrates the therapeutic potential of cell-based tissue-engineered constructs to restore whole-muscle biomechanics for future clinical treatment of VML. Whole-muscle biomechanical analysis aids understanding of VML pathophysiology and evaluation of tissue-engineered treatment strategies. Such preclinical studies may reveal key principles to inform the rational design of individualized muscle regeneration strategies to restore muscle structure and function based on clinical imaging and computational modeling.^10^

Though MuSCs and muscle-derived cell lines differentiate readily into myotubes which can undergo visible twitches, the maturation of engineered muscle fibers to generate sustained active stress is a persistent challenge. With the bioconstruct treatment, maximum tetanic force increased on a scale that was measureable in the whole muscle.^5^ The newly formed muscle tissue cannot be isolated from the rest of the uninjured muscle to test its force generation directly. Our observation that the treated muscles show a proportional increase in force and mass is consistent with the formation of new fibers with mature stress generation. Conversely, the absence of the cellular component in the treatment failed to restore force and mass.

Activities of daily living require muscles to generate active force over the range of motion of the joint, as determined by muscle fiber architecture and measured by the force-length curve. Volumetric muscle loss alters the force-length curve, as observed in patients,^11^ a previous animal model,^12^ and in the present study. The treatment with cells but not with scaffold only restored the width of the force-length curve, consistent with remodeling of fiber architecture. For the treatment, we used cells that were as close to the native state in their physiological niche as possible (freshly isolated from healthy muscle), and delivered them into the mechanical and biochemical environment into which they must form a muscle. The forces, strains, and aligned substrate experienced by the cells in the implanted decellularized muscle scaffold can all affect fiber formation,^2,7,13^ and thereby also contribute to whole-muscle architecture restoration. The restoration of the width of the force-length curve suggests that newly formed fibers have similar length to the native fibers. The shorter muscle belly length at which optimal force occurs suggests a difference in the architectural organization of the fibers within the muscle belly. Our results support the requirement of de novo formed fibers in repairing VML injuries, suggesting that cellularized scaffold treatments might be more effective than decellularized treatments, consistently with previous reports.^5,14^ In the future, incorporating micro- and macro- scale features that mimic the endogenous niche to cue the restoration of muscle fibers within the restored architecture is warranted.^15^

Scarring is a challenge for treating VML in patients. The increased passive force in the injured group without treatment is consistent with increased fibrotic tissue seen in histology^5^ and with the increased passive tension in a previous rat TA model of VML.^12^ Increased fibrosis might also contribute to explain the increased half-relaxation time, possibly due to a “spring-like” return of energy that follows the active contraction. Indeed, this resulting passive tension could facilitate the return to the initial muscle length. However, it would also offer increased resistance during the active contraction, which might participate in reducing the force generation. We showed previously^5^ and here that cell-based tissue engineering treatment reduces fibrosis in a TA model of VML. These observations are consistent with previous reports that showed how muscle stem cells and myogenic progenitors interact in their niche with other cell types, in particular with fibrogenic cells, to actively regulate their extracellular environments.^16^ The decrease in passive force, back to uninjured levels, is promising for the therapeutic potential of our treatment to reduce scarring. A challenge for the future is to understand the formation and remodeling of the extracellular matrix in VML and in tissue-engineered repair.

## Methods

Animal protocols were carried out in accordance with Stanford University and VA animal care and use guidelines. Cell sorting, bioconstruct preparation, VML injury, and bioconstruct transplantations were performed previously described,^5^ briefly summarized below.

### Animals

C57BL/6, mice were obtained from Jackson Laboratory. All experimental mice employed were 3–6 months old. Mice were housed and maintained in the Veterinary Medical Unit at the Veterans Affairs Palo Alto Health Care Systems. Animal protocols were approved by the Administrative Panel on Laboratory Animal Care of Stanford University.

### Primary cell sorting and bioconstruct preparation

Bioconstructs were prepared by injecting primary cells into scaffolds. We isolated primary cells from skeletal muscles of 3-months-old wild-type male mice (C57Bl/6, Jackson Laboratories) using chemical digestion followed by fluorescence-activated cell sorting. We collected satellite cells, endothelial cells, hematopoietic cells, and fibro-adipogenic progenitors. A mixture was made by combining cells with extracellular matrix proteins to form a hydrogel. Evans blue dye was included in the mixture to enable visualization during injection into the scaffold. Scaffolds were mouse tibialis anterior (TA) muscles that were decellularized with a series of detergents. The mixture was injected into the scaffold using a syringe pump.

### VML injury and transplantation surgery

Host mice (NOD/SCID, Taconic) were divided into four treatment groups. The uninjured control group received no-VML injury or treatment. The untreated VML group was injured as previously described.^5^ Briefly, VML injury was performed by removing rectangular piece of muscle tissue (~15 mg) from the TA. The skin was sutured closed over the wound. For the treatment groups (scaffold only or scaffold plus cells), bioconstructs were generated respectively with scaffold only or with scaffold plus cells as previously described.^15^ The two treatment groups were injured the same way, and the bioconstructs were sutured into the wound with two proximal sutures and one distal suture into the tendon. The medial and lateral edges of the muscle were sutured closed over the bioconstruct. The skin was sutured closed over the muscle. Surgeries for muscle ablation and bioconstruct implantation were performed blinded by different investigators. The bioconstruct fabrication procedure is standardized by the use of micropumps and micromanipulators.

### In vivo force measurement

One month after surgery (age-matched for the uninjured control group), forces in the TA muscles were measured in vivo and ex vivo. For in vivo measurements, mice were anesthetized and the sciatic nerves were placed into bipolar nerve cuffs made with stainless steel wire. To access the sciatic nerve, an incision was made in the skin on the lateral surface of the upper leg. The fascia connecting the hamstrings and vastus lateralis was clipped, and the muscles were retracted to reveal the sciatic nerve. A strip of parafilm was placed under the nerve and stimulator to electrically isolate the surrounding muscles from the stimulator.

The distal tendon of the TA muscle was attached to a force transducer (Aurora Scientific) using suture and a stainless steel wire. The tendon was sutured to one end of the wire and the force transducer was attached to the other end of the wire. To prevent suture slippage during active muscle force, the tendon was first sandwiched between two pieces of wooden toothpick with superglue. The suture was knotted around the tendon proximal to the wooden pieces and then knotted onto a hook at the end of the wire. No suture loops or other significant sources of compliance were present in the system.

We measured viscoelastic force relaxation, passive force, and active twitch force across a range of muscle lengths. A step increase in length of 0.5 mm was applied to the muscle, and the viscoelastic force relaxation trace was recorded for 15 s. We fit a two-phase decay equation to the force relaxation curve and found time constants *τ*_1_ and *τ*_2_ at each length:

$$P = P_0 + P_1{\mathrm{e}}^ \wedge ( - \tau ^ \wedge - \tau _1) + P_2{\mathrm{e}}^ \wedge ( - \tau ^ \wedge - \tau _2).$$

The measurement was repeated at each muscle length. The mean value of *τ*_1_ and *τ*_2_ across trials at different muscle lengths is reported for each TA.

After 1 min of relaxation, bipolar twitch stimuli were applied via the nerve cuff (6 twitches, 1 Hz, 1 ms stimulus duration). The baseline force before each twitch was subtracted from the maximum force during the twitch to calculate active twitch force. The three highest twitch forces were averaged at each length. The baseline force before the final twitch was recorded as the passive force at that length. Thus we measured active and passive forces across a range of lengths in 0.5 mm increments. The three maximal twitches at the optimal length were further analyzed for time from stimulus to peak force and time to relax from peak force to half of the peak force. The force-length curve was normalized to maximum twitch force and offset to length at maximum twitch force.

After completing the length-tension curve with twitches (2 increments of 0.5 mm beyond the optimal), the muscle was returned to optimal length and stimulated at increasing frequencies (60, 80, 100, 120, and 140 Hz) to determine the maximum tetanic force. Maximum twitch force was plotted against muscle mass as an indirect measurement of active specific tension. Length at optimal force was measured with digital calipers as the distance from the fibular head to the muscle-tendon junction.

Both TAs were tested. After the first TA was tested, the lower leg was wrapped in parafilm with PBS while the contralateral TA was tested. Limb order was randomized. Preliminary testing in C57Bl6 mice showed no difference between TAs tested first or second. An Aurora Scientific 1300-A Whole Mouse Test System was used to gather force production data. Animals were killed by cervical dislocation under anesthesia.

### Ex vivo force measurement

To measure the force independent of innervation, we isolated the TA in a bath of oxygenated Ringer’s solution and stimulated it with plate electrodes. Immediately after euthanasia, the distal tendon of the TA, the TA, and the knee (proximal tibia, distal femur, patella, and associated soft tissues) were dissected out and placed in Ringer’s solution (Sigma) maintained at 25 °C with bubbling oxygen with 5% carbon dioxide. The proximal tibia was sutured to a rigid wire attached to the force transducer and the distal tendon was sutured to a rigid fixture. No suture loops or slack was present in the system. The contralateral limb was immediately dissected and kept under low passive tension in oxygenated Ringer’s solution bath until measurement. Supramaximal stimulation voltage was found and the active force-length curve was measured in a manner similar to the in vivo condition. After measurement, the muscle was dissected free and the mass measured. An Aurora Scientific 1300-A Whole Mouse Test System was used to gather force production data.

### Image analysis

Image J was used to calculate the percentage of area composed of Collagen by using the color threshold plugin to create a mask of only the area not occupied by transplanted scaffold and positive for Collagen. That area was then divided over the total area of the sample which was found using the free draw tool.

### Statistical analysis

A second-order regression curve was fit to the in vivo and ex vivo length-tension curves of each group and the second-order term compared with an *f*-test. The passive force-length relationship was compared with linear regression between the lengths of L_o_ −0.5 to L_o_ +0.5. The active and passive curves of the VML group treated with scaffold plus cells were pairwise compared to those from each of the other groups. Differences among the means of optimal length, time to peak twitch force, time from twitch peak to half relaxation, and viscoelastic time constants were compared with one-way ANOVA with post hoc Dunnett pairwise comparison between the VML group treated with scaffold plus cells and each of the other groups. To evaluate potential differences in the active stress generation among the groups, we plotted maximum ex vivo tetanic force vs mass and used linear regression to test the null hypothesis that one line describes all the groups. To analyze collagen area quantification data, two-sided test was used.

### Immunostaining

A 1-h blocking step with 20% donkey serum/0.3% Triton in PBS was used to prevent unwanted primary antibody binding for all samples. Primary antibodies were applied and allowed to incubate over night at 4 °C in 20% donkey serum/0.3% Triton in PBS. After 4 washes with 0.3% PBST, fluorescently conjugated secondary antibodies were added and incubated at room temperature for 1 h in 0.3% PBST. After three additional rinses each slide was mounted using Fluoview mounting media.

### Imaging

Samples were imaged using standard fluorescent microscopy (Zeiss Observer Microscope Inverted Motorized Fluorescence Phase Contrast) and either a ×10 or ×20 air objective. Volocity imaging software was used to adjust excitation and emission filters and came with pre-programmed AlexaFluor filter settings which were used whenever possible. All exposure times were optimized during the first round of imaging and then kept constant through all subsequent imaging.

### Antibodies

The following antibodies were used in this study. The source of each antibody is indicated: Collagen I (Cedarlane Labs, #CL50151AP, 1:200); Laminin (Millipore, #MAB1903, 1:750).

### General methods

Unless stated otherwise, sample size (*n*-values) are reported as biological replicates of mice and/or SC isolations from separate mice performed on different days. In most cases, the data presented were compiled over the course of 3 years, as mice with the appropriate genotype became available. Therefore, the magnitude of the effect and variability in the measurements were primary factors in determining sample size and replication of data. Although samples were not explicitly randomized or blinded, mouse identification numbers were used as sample identifiers and thus the genotypes and experimental conditions of each mouse/sample were not readily known or available to the experimenters during sample processing and data collection. The only criteria used to exclude samples involved the health of the animals, such as visible wounds from fighting. In these cases, the animals were handled in accordance with approved IACUC guidelines.

The research was conducted in accordance to all relevant guidelines and procedures

## Data Availability

Raw data generated are available from the corresponding author (TAR) on reasonable request.
